# Causal effect of gut microbiota on the risk of prostatitis: a two-sample Mendelian randomization study

**DOI:** 10.1007/s11255-024-04020-w

**Published:** 2024-04-04

**Authors:** Dalu Liu, Yangyang Mei, Nuo Ji, Bo Zhang, Xingliang Feng

**Affiliations:** 1 Department of General Surgery, The Affiliated Chuzhou Hospital of Anhui Medical University, Chuzhou, Anhui China; 2 Department of Urology, Jiangyin People’s Hospital of Jiangsu Province, Jiangyin, Jiangsu China; 3 Department of Urology, The First People’s Hospital of Changzhou, Changzhou, Jiangsu China; 4 Department of Urology, The Third Affiliated Hospital of Soochow University, Changzhou, Jiangsu China

**Keywords:** Gut microbiota, Chronic prostatitis, Mendelian randomization, Causality, Gut–prostate axis, Genome-wide association study

## Abstract

**Background:**

Recent studies demonstrated that chronic prostatitis (CP) is closely related to the gut microbiota (GM). Nevertheless, the causal relationship between GM and CP has not been fully elucidated. Therefore, the two-sample Mendelian randomization (MR) analysis was employed to investigate this association.

**Methods:**

The summary data of gut microbiota derived from a genome-wide association study (GWAS) involving 18,340 individuals in the MiBioGen study served as the exposure, and the corresponding summary statistics for CP risk, representing the outcome, were obtained from the FinnGen databases (R9). The causal effects between GM and CP were estimated using the inverse-variance weighted (IVW) method supplemented with MR-Egger, weighted median, weighted mode, and simple mode methods. Additionally, the false discovery rate (FDR) correction was performed to adjust results. The detection and quantification of heterogeneity and pleiotropy were accomplished through the MR pleiotropy residual sum and outlier method, Cochran’s *Q* statistics, and MR-Egger regression.

**Results:**

The IVW estimates indicated that a total of 11 GM taxa were related to the risk of CP. Seven of them was correlated with an increased risk of CP, while the remained linked with a decreased risk of CP. However, only Methanobacteria (OR 0.86; 95% CI 0.74–0.99), Methanobacteriales (OR 0.86; 95% CI 0.74–0.99), NB1n (OR 1.16; 95% CI 1.16–1.34), Methanobacteriaceae (OR 0.86; 95% CI 0.74–0.99), Odoribactergenus Odoribacter (OR 1.43; 95% CI 1.05–1.94), and Sutterellagenus Sutterella (OR 1.33; 95% CI 1.01–1.76) still maintain significant association with CP after FDR correction. Consistent directional effects for all analyses were observed in the supplementary methods. Subsequently, sensitivity analyses indicated the absence of heterogeneity, directional pleiotropy, or outliers concerning the causal effect of specific gut microbiota on CP (*p* > 0.05).

**Conclusion:**

Our study demonstrated a gut microbiota–prostate axis, offering crucial data supporting the promising use of the GM as a candidate target for CP prevention, diagnosis, and treatment. There is a necessity for randomized controlled trials to validate the protective effect of the linked GM against the risk of CP, and to further investigate the underlying mechanisms involved.

**Supplementary Information:**

The online version contains supplementary material available at 10.1007/s11255-024-04020-w.

## Introduction

Chronic prostatitis or chronic pelvic pain syndrome (CP/CPPS), also termed National Institutes of Health (NIH) category III prostatitis, is one of the most prevalent conditions affecting men under the age of 50 years, with reported prevalence rates ranging between 8.4 and 14% [1, 2]. CP/CPPS is characterized by pelvic or perineal pain, lower urinary tract symptoms, and sexual dysfunction [3]. Although the pathogenesis of CP/CPPS is still not fully understood, a variety of causes, including inflammation‐mediated abnormal pelvic floor neuromuscular activity, dysfunction of lower urothelial cells, immune abnormalities, neuroendocrine abnormalities, and psychological factors, are believed to be involved [4]. Due to diverse clinical presentations and complex pathogenesis of CP/CPPS, there is currently lacking of effective therapeutic options for CP/CPPS patients that would enable a more rational-driven therapy. Consequently, it is of great necessity and importance to conduct more in‐depth research to investigate the underlying mechanisms of CP/CPPS, providing basis to explore novel and efficacious treatments.

The gut microbiota is a vast and intricate community of microbial species that reside in the human gastrointestinal tract, playing a pivotal role in human health and diseases [5]. The human gut microbiota can impact host physiology by regulating various processes, including inflammation, oxidative stress, immune function, and anabolic balance [6, 7]. On the contrary, the physiological or pathological status of the host can also affect the abundance and functionality of the gut microbiota [8]. Growing evidence suggests that CP/CPPS is closely correlated with altered gut microbiota [9]. According to a recent study by Wang et al., the gut microbiota diversity bacterial species abundance with CP/CPPS was significantly different from those of healthy controls [10]. Specifically, Shoskes et al. demonstrated that lower counts of Prevotella may serve as both disease biomarker and potential therapeutic target in CPPS [11]. Therefore, it can be reasonably speculated that gut microbiota may participate in the regulation of CP through the microbiome–gut–prostate axis. However, all these results were mainly based on the observational and cross-sectional studies, which included small number of participants and confounding factors [12]. Therefore, the causal relationship between gut microbiota and sleep disturbances remains unclear, which need to be furtherly explored.

Mendelian randomization (MR) is a genetic epidemiological approach that deduces causality between exposure and outcome using genetic variants to compose instrumental variables (IVs) [13]. It is grounded in the concept that single-nucleotide polymorphisms (SNPs) undergo random variation and distribution during gamete formation, remaining unaffected by confounding factors after gametogenesis [14]. Consequently, the MR study could effectively circumvent the confounding bias and reverse causality, which often seen in the traditional epidemiological studies [15]. The single-nucleotide polymorphisms (SNPs) from the most recent genome-wide association studies (GWASs) were utilized as instrumental variables. To our knowledge, no MR research was performed to investigate the association between gut microbiota and CP.

In this study, we explored the potential causal relationship between 211 gut microbiota taxa and CP by MR study design, using summary-level statistics of the genome-wide association study (GWAS) from the MiBioGen and FinnGen consortia. The findings of this research can serve as a foundation for illuminating potential mechanisms underlying the development of CP in relation to the characteristics of the gut microbiota.

## Materials and methods

### Study design

**Fig. 1:**
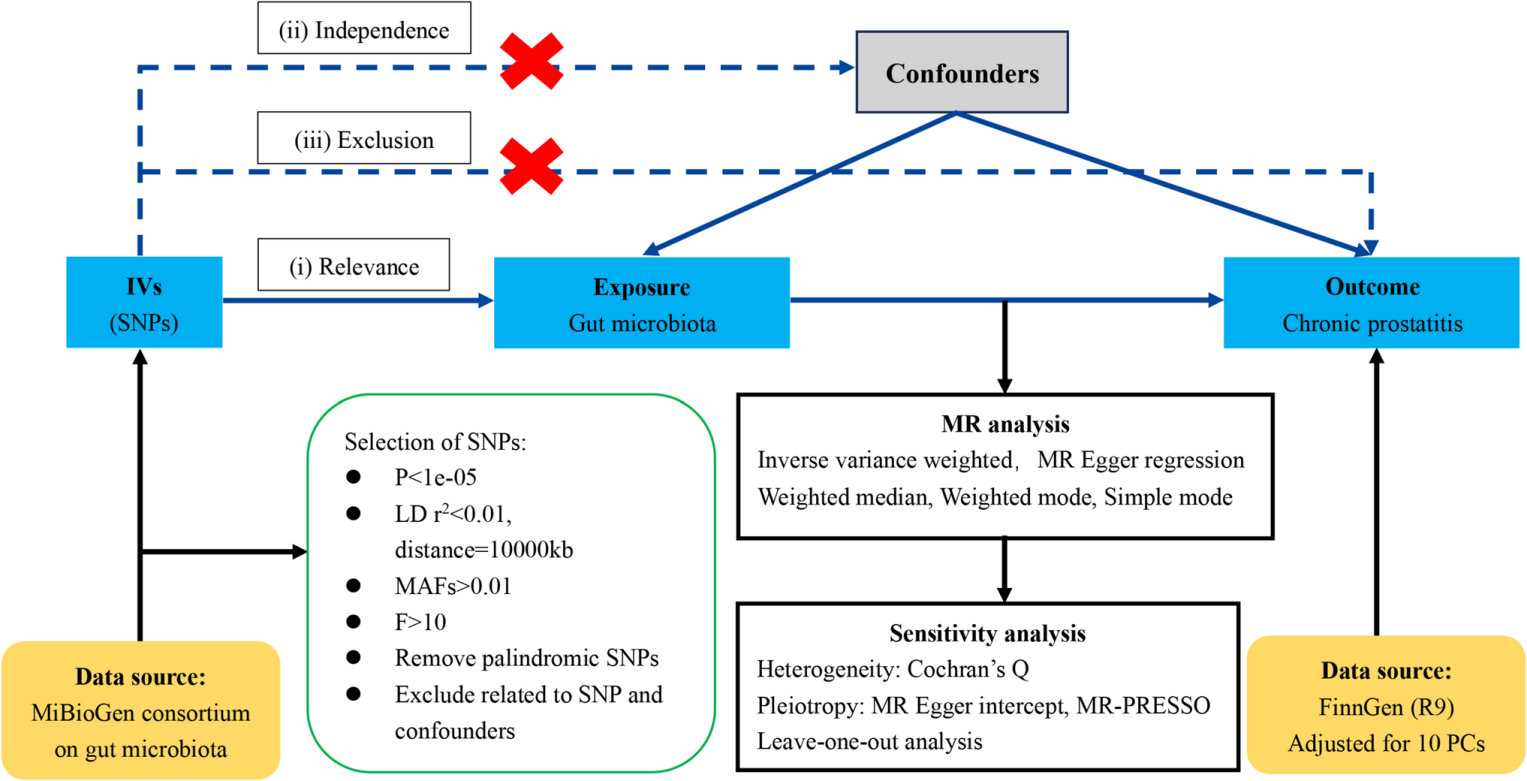
Flowchart of MR study design and MR core assumptions. *MR* Mendelian randomization, *GWAS* genome-wide association study, *SNPs* single-nucleotide polymorphisms, *IVW* inverse-variance weighted, *LD* linkage disequilibrium, *MR-PRESSO* MR pleiotropy residual sum and outlier

The two-sample MR study was performed in accordance with the STROBE-MR guidelines [16] (Supplementary Table S1). All data utilized for the MR analysis were sourced from the publicly available GWASs with participant consent and ethical approvals, and no further ethical approval was needed for our study. We used GWAS summary data derived from different consortia to avoid sample overlap, the exposure of gut microbiota obtained from the MibioGen consortium [17] and the outcome of prostatitis obtained from the FinnGen Project. Additionally, we restricted the exposure and outcome data to individuals of European ancestry to mitigate potential bias. There were three core assumptions that need to be satisfied when conducting MR analysis: (i) IVs are robustly associated with the exposure data; (ii) IVs are independent of potential confounders; (iii) IVs affect outcome solely through the exposure of interest [18]. To determine whether the gut microbiota contributes to the prevention or promotion of CP, 211 gut microbiota taxa were selected as exposure of interest, and CP was defined as outcome of interest. Multiple MR methods were used for statistical analysis, and several sensitivity analyses (the heterogeneity test, the pleiotropy test, and leave-one-out analysis) were performed sequentially to confirm the robustness of our results. The concise flowchart for our MR analysis is depicted in Fig. 1.

### Data sources

The summary statistics of intestinal bacteria were accessed from the largest GWAS meta-analysis of the human microbiome published by the MibioGen consortium. The study involved 18,340 participants from 24 cohorts, utilizing whole-genome genotyping data and 16S rRNA genes extracted from fecal microbiomes. Among these cohorts, most of them are European descent (*N* = 13,266). The microbial composition was characterized by focusing on three distinct variable regions of the 16S rRNA gene: V4, V3–V4, and V1–V2. Taxonomic classification was carried out using direct taxonomic binning. Subsequently, microbiota quantitative trait loci (mbQTL) mapping analysis was conducted to identify host genetic variants associated with the abundance of gut bacterial taxa [17]. The lowest taxonomic classification of the study was at genus level. The GWAS finally identified a total of 211 GM taxa, including 9 phyla, 16 classes, 20 orders, 35 families, and 131 genera. However, only 196 GM taxa were finally incorporated in the current MR analysis, of which 15 GM taxa were excluded without specific species names. GWAS summary data for CP were obtained from the FinnGen consortium R9 release dataset (https://r9.finngen.fi/), encompassing 3760 cases and 119,297 controls. In this database, the research endpoints were delineated based on ICD-10 codes, and all data were adjusted via SAIGE, using sex, age, genotyping batch and ten principal components as covariates [19].

### Instrumental variables

To ensure the precision of the MR analysis regarding the causal effects of gut microbiota in the risk of CP, a series of quality inspection procedures was systematically employed during the selection of genetic predictors associated with microbiome characteristics, which also remained consistent with the MR core assumptions. Similar to most current MR studies, we initially adopted a genome-wide significance threshold (*P* < 5 × 10^−8^) for SNPs screening. Due to the limited number of SNPs meeting genome-wide significance, we utilized SNPs with a more lenient threshold (*P* < 1 × 10^−5^) as potential IVs for each genus [20]. To minimize the effects of linkage disequilibrium (LD), we performed LD clumping using a 10 MB clumping window and an *R*^2^ threshold of < 0.001, based on European ancestry reference data from the 1000 Genomes Project. We excluded the SNPs with minor allelic frequencies (MAFs) ≤ 0.01. Additionally, to prevent bias resulting from the employment of weak instruments, F-statistics were computed for each SNP to assess statistical strength. Only robust IVs with *F*-statistics exceeding 10 for each of our exposures of interest were retained [21]. The *F*-statistics were calculated based on the formula: *F* = (*R*^2^/1 − *R*^2^)*(N-K-1) [22]. In the formula, *R*^2^ represents the variance explained by each IV calculated by the formula (*R*^2^ = 2*(1 − MAF)*MAF*β^2^) [23], N represents the sample size, K represents the number of IVs, and MAF stands for minor allele frequency. Meanwhile, ambiguous and palindromic SNPs, for which the effects could not be rectified during the harmonization process, were excluded. In reverse MR analysis, the genome-wide significance threshold for exposure data (CP) was established at *p* < 5 × 10^−8^; the remaining criteria and parameters remained consistent with forward MR.

Furthermore, to satisfy the third MR assumption, all eligible IVs were scanned using the PhenoScanner V2, a database of human genotype–phenotype associations, to detect whether these IVs were correlated with the potential risk factors, including smoking, alcohol consumption, and body mass index (BMI) [24]. Subsequently, we excluded SNPs associated with any of these potential confounders.

### Statistical analysis

To scrutinize the hypothesized causal association between gut microbiota and CP by MR analysis, multiple statistical models were employed, comprising Inverse Variance Weighted (IVW), MR Egger, Weighted Median, Weighted Mode, and Simple Mode. The IVW estimate is a weighted linear regression model with the intercept set to zero [25]. It stands as the most frequently employed and crucial calculation method in MR studies. However, it is worth noting that the IVW would be influenced by the IV pleiotropy and heterogeneity. In devoid of horizontal pleiotropy and heterogeneity, IVW could provide the most accurate estimate even when the four supplementary methods yield negative results [26]. Consequently, the MR analysis results were based on the IVW, and the results were shown as the odds ratio (OR) and 95% confidence interval (CI). MR-Egger allows all genetic variants to exhibit pleiotropic effects but necessitates that these pleiotropic effects are independent of the variant–exposure association [27]. The weighted median method enables accurate estimation of causal associations even when up to 50% of IVs are deemed invalid [28]. When the majority of individual SNP causal effect estimates are derived from efficient SNPs, the weighted model remains consistent even in the presence of invalid SNPs [28]. As for simple mode, it is an unweighted mode of the empirical density function of causal estimation [29].

Sensitivity analysis has been conducted to identify potential underlying pleiotropy and heterogeneity, as these factors can significantly impact MR estimates. The Cochrane’s *Q* tests derived from the IVW and MR-Egger methods were performed to detect the heterogeneity among IVs, and no heterogeneity was observed when the *p* value < 0.05 [30]. The intercept of the MR-Egger regression was computed to evaluate horizontal pleiotropy, with a p value greater than 0.05 indicating a weak possibility of pleiotropic effects in the causal analysis [31]. It is worth noting that we would discard the causal inference in the absence of horizontal pleiotropy. Additionally, MR-Pleiotropy Residual Sum and Outlier Methods (MR-PRESSO) were also employed to evaluate the overall horizontal pleiotropy, and identify the abnormal SNPs which led to the pleiotropy [32]. In cases where outliners were detected, the MR analysis was reiterated after removing these specific SNPs. Meanwhile, a leave-one-out analysis was conducted to visually evaluate whether the MR estimates were biased by any single SNP, by sequentially omitting each SNP. To infer causal direction, the MR Steiger directionality test was administered in the MR analysis [33]. The causal link is considered directionally credible if the IVs explain more variance in the exposure than in the outcome. Finally, we also tried to perform the reverse MR analysis of CP and gut microbiota. During the process, the SNPs strongly associated with CP were employed as IVs, with the positive gut microbiota identified in the forward MR analysis serving as outcome. However, we failed to perform the reverse MR analysis due to insufficient eligible SNPs, despite the relaxation of the IV selection criteria. Consequently, no additional reverse MR analysis results were presented in the results section.

The statistical power of the MR estimates was assessed using an online calculator tool (http://cnsgenomics.com/shiny/mRnd/) provided by Stephen Burgess [34]. The computation of MR estimates necessitates the inclusion of *R*^2^ summations for each SNP, which denotes the fraction of variability in exposure that can be explained by genetic variation. To address multiple hypothesis testing, the Benjamini and Hochberg false discovery rate (FDR) correction was performed to adjust our results [35]. The FDR adjusted *p* value of < 0.05 was considered statistically significant. The *R* packages “TwoSampleMR” and “MRPRESSO” were applied to carry out our statistical analysis including MR analysis and sensitivity analysis using the publicly available R software (version 4.1.2).

## Results

### Selection of instrumental variables

Initially, 14,587 SNPs associated with the gut microbiota were identified as IVs from the MiBioGen Consortium, employing a relatively lenient significance threshold (*p* < 1 × 10^–5^). This set encompassed 211 bacterial traits, comprising 131 genera, 35 families, 20 orders, 16 classes, and 9 phyla. Following a series of quality control measures, a total of 2036 SNPs were ultimately included in the analysis. Moreover, all IVs demonstrated *F*-statistics greater than 10, indicating an absence of evidence for weak instrument bias (Supplementary Table S2).

### Results of MR analysis

**Fig. 2:**
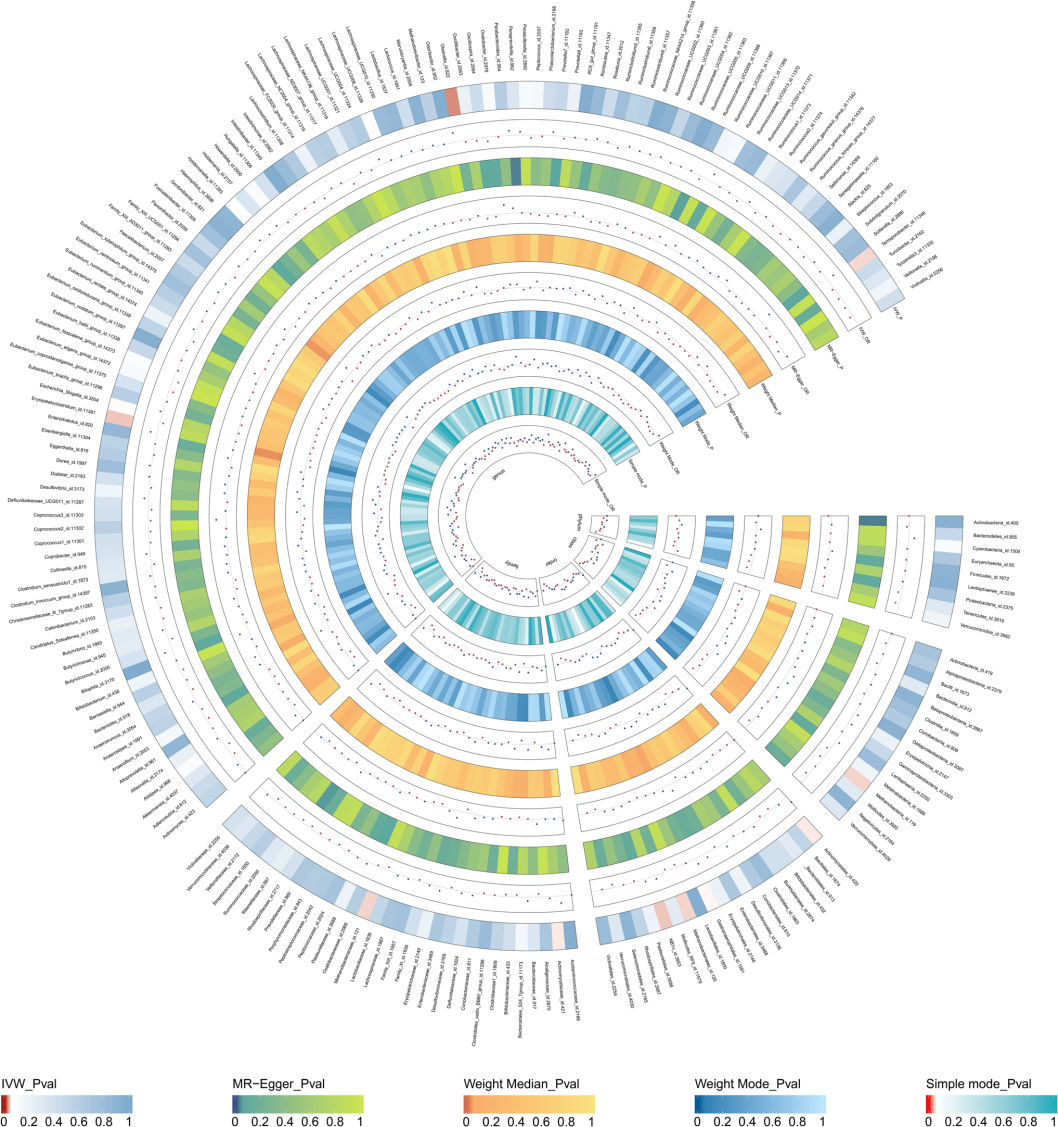
The circus plot showing the MR result of all gut microbiota. *IVW* inverse-variance weighted, *OR* odds ratio

**Fig. 3:**
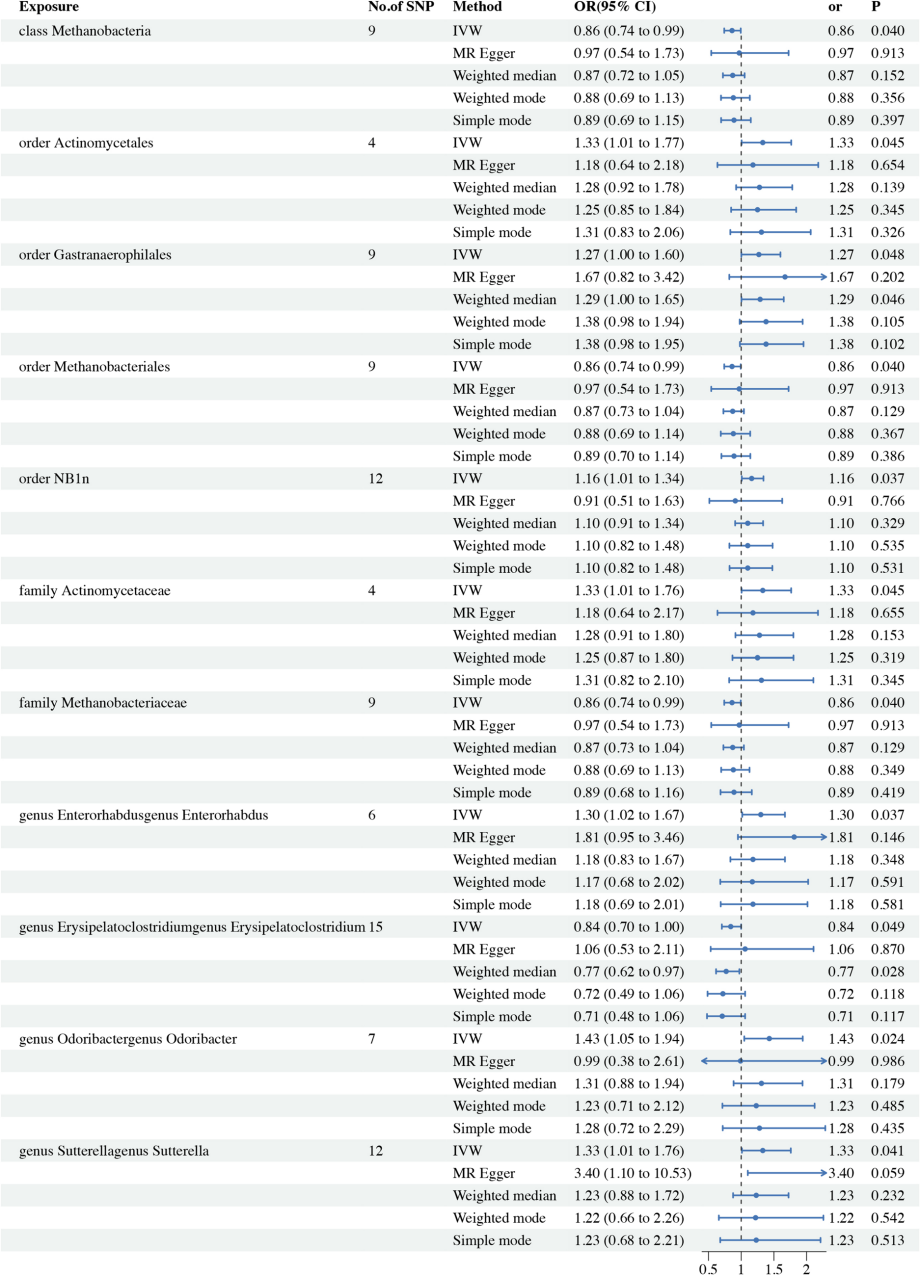
Significant results of the associations between genetically predicted gut microbiota with risk of CP. *IVW* inverse-variance weighted, *CP* chronic prostatitis, *OR* odds ratio, *CI* confidence interval

**Fig. 4:**
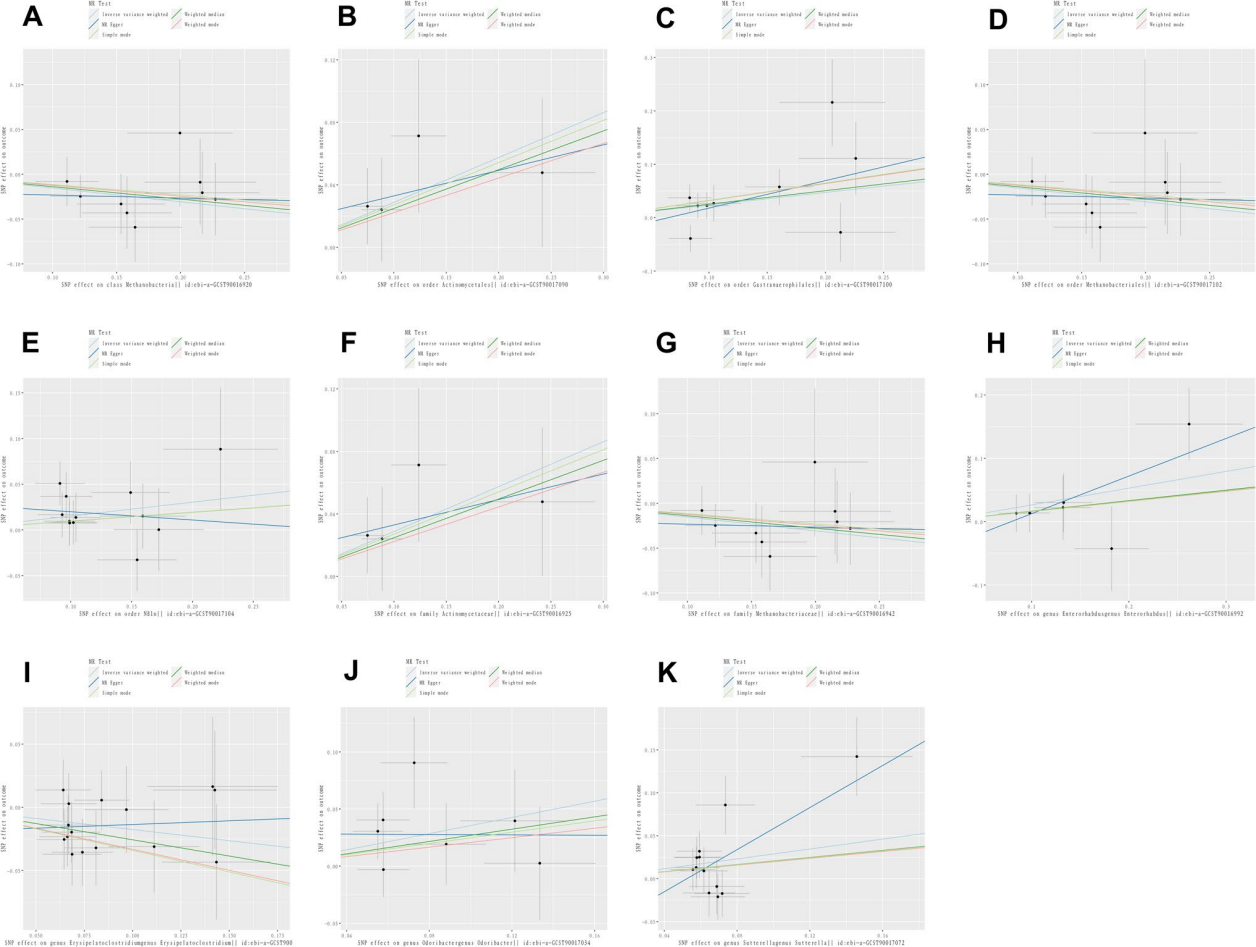
Scatter plots of the causal effect of gut microbiota on risk of CP. **A** class Methanobacteria; **B** order Actinomycetales; **C** order Gastranaerophilales; **D** order Methanobacteriales; **E** order NB1n; **F** family Actinomycetaceae; **G** family Methanobacteriaceae; **H** genus Enterorhabdusgenus Enterorhabdus; **I** genus Erysipelatoclostridiumgenus Erysipelatoclostridium; **J** genus Odoribactergenus Odoribacter; **K** genus Sutterellagenus Sutterella. The slope of the line represents the causality of the different MR methods

MR analyses were conducted for each pair of exposure (bacterial taxa) and outcome (CP) to investigate causal associations, employing five MR methods (IVW, MR Egger, simple mode, weighted median, and weighted mode) (Fig. 2). The results reaching the threshold of *p* < 0.05 according to the IVW method are depicted in Fig. 3. The OR, representing an elevated risk of CP per standard deviation increase in gut microbiota feature abundance, was used to quantify the causal effects. IVW analyses showed suggestive causal effects, where genetically predicted increased abundance of Methanobacteria at the class level (OR 0.86; 95% confidence interval [CI] 0.74–0.99; *p* = 0.040) and Methanobacteriales at the order level (OR 0.86; 95% CI 0.74–0.99; *p* = 0.040) had protective effects on CP risk. Conversely, host-genetic-driven increased Actinomycetales (OR 1.33; 95% CI 1.01–1.77; *p* = 0.045), Gastranaerophilales (OR 1.27; 95% CI 1.00–1.60; *p* = 0.048), and NB1n (OR 1.27; 95% CI 1.00–1.60; *p* = 0.048) at the order level was associated with a higher risk of CP. Suggestive associations at the family level were also observed, with Actinomycetaceae (OR 1.33; 95% CI 1.01–1.76; *p* = 0.045) positively linked to CP risk, while Methanobacteriaceae at the family level (OR 86; 95% CI 0.74–0.99; *p* = 0.040) was negatively correlated with CP risk. At the genus level, erysipelatoclostridiumgenus Erysipelatoclostridium (OR 0.84; 95% CI 0.70–1.00; *p* = 0.049) showed a negative correlation with risk of CP. Three genera were positively correlated with risk of CP, namely, Enterorhabdusgenus Enterorhabdus (OR 1.30, 95% CI 1.02–1.67, *p* = 0.037), Odoribactergenus Odoribacter (OR 1.43, 95% CI 1.05–1.94, *p* = 0.024), and Sutterellagenus Sutterella| (OR 1.33, 95% CI 1.01–1.76, *p* = 0.041) (Table 1). The results from other complementary analytical methods were consistent in direction with the primary analysis, enhancing confidence in the true causal association (Supplementary Table S3, Fig. 3). The scatter plot visually represents the causal effects between gut microbiota and CP (Fig. 4). However, only Methanobacteria, Methanobacteriales, NB1n, Methanobacteriaceae, Odoribactergenus Odoribacter, and Sutterellagenus Sutterella showed significant associations after the FDR correction (*p* < 0.05) (Supplementary Table S3).

**Table 1:** Positive MR results of casual links between gut microbiota and chronic prostatitis. (p < 1e-05)

Taxa	nSNPs	Method	OR (95%CI)	*p* value	Horizontal pleiotropy			Heterogeneity		MR-PRESSO
					Egger intercept	SE	*p* value	Cochran’s Q	*p* value	*p* value
Class Methanobacteria	9	IVW	0.86 (0.74, 0.99)	0.040				2.48	0.96	0.207
		MR Egger	0.97 (0.54, 1.73)	0.913	− 0.020	0.047	0.686	2.30	0.94	
Order Actinomycetales	4	IVW	1.33 (1.01, 1.77)	0.045				0.77	0.86	0.836
		MR Egger	1.18 (0.64, 2.18)	0.654	0.017	0.037	0.701	0.58	0.75	
Order Gastranaerophilales	9	IVW	1.27 (1.00, 1.60)	0.048				13.47	0.10	0.406
		MR Egger	1.67 (0.82, 3.42)	0.202	− 0.033	0.041	0.446	12.32	0.09	
Order Methanobacteriales	9	IVW	0.86 (0.74, 0.99)	0.040				2.48	0.96	0.991
		MR Egger	0.97 (0.54, 1.73)	0.913	− 0.020	0.047	0.686	2.30	0.94	
Order NB1n	12	IVW	1.16 (1.10, 1.34)	0.037				7.46	0.76	0.507
		MR Egger	0.91 (0.51, 1.63)	0.766	0.029	0.034	0.417	6.75	0.75	
Family Actinomycetaceae	4	IVW	1.33 (1.01, 1.76)	0.045				0.78	0.86	0.836
		MR Egger	1.18 (0.64, 2.17)	0.655	0.017	0.037	0.700	0.58	0.75	
Family Methanobacteriaceae	9	IVW	0.86 (0.74, 0.99)	0.040				2.48	0.96	0.990
		MR Egger	0.97 (0.54, 1.73)	0.913	− 0.020	0.047	0.686	2.30	0.94	
Genus Enterorhabdusgenus Enterorhabdus	6	IVW	1.30 (1.02, 1.67)	0.037				4.41	0.49	0.315
		MR Egger	1.81 (0.95, 3.46)	0.146	0.047	0.043	0.339	3.23	0.52	
Genus Erysipelatoclostridiumgenus Erysipelatoclostridium	15	IVW	0.84 (0.70, 1.00)	0.049				6.72	0.94	0.828
		MR Egger	1.06 (0.53, 2.11)	0.870	0.019	0.028	0.501	6.24	0.94	
Genus Odoribactergenus Odoribacter	7	IVW	1.43 (1.05, 1.94)	0.024				5.37	0.50	0.387
		MR Egger	0.99 (0.38, 2.61)	0.986	0.028	0.036	0.472	4.77	0.44	
Genus Sutterellagenus Sutterella|	12	IVW	1.33 (1.01, 1.76)	0.041				15.80	0.15	0.156
		MR Egger	3.40 (1.10, 10.53)	0.059	0.064	0.038	0.126	12.36	0.26	

*OR* odds ratio, *CI* confidence interval, *IVW* inverse-variance weighted, *SNP* single-nucleotide polymorphism, *MR* Mendelian randomization, *SE* standard error, *MRPRESSO* MR-Pleiotropy Residual Sum and Outlier

### Results of sensitivity analysis

Sensitivity analyses were conducted to ensure the robustness of the results. Cochran’s *Q* revealed homogeneity among the instrumental variables. The results of the MR-Egger regression indicated no evidence of pleiotropic effects (all *P* intercept > 0.05), along with the MR-PRESSO global test (all *P* global test > 0.05). This suggests that instrumental variables are unlikely to influence CP risk through pathways other than the gut microbiome, as indicated by significant results. Detailed results can be found in Table 1. Besides, no outliers were detected through MR-PRESSO analyses. Supplementary Fig. S2 provide a summary of the causal effects of significant taxa on chronic prostatitis in the leave-one-out analysis. Moreover, the leave-one-out analysis and the forest plots did not identify any SNP that significantly altered the overall results, ensuring the stability of the outcomes (Supplementary Fig. S3). Furthermore, our study possessed satisfactory power (more than 80%) to evaluate the causal effects of these gut microbiome features on CP (Supplementary Table S4).

## Discussion

In the present MR study, we are the first to demonstrate a causal effect of the gut microbiota on the prostate through large-scale GWAS summary data. Our results showed a causal relationship between 11 gut microbiota and CP. After FDR correction, the genetically predicted Methanobacteria, Methanobacteriales, NB1n, Methanobacteriaceae, Odoribactergenus Odoribacter, and Sutterellagenus Sutterella still have significant causal effects on CP. Modulation of specific bacterial traits may help CP-related symptoms.

The gut microbiota controls multiple pathways involved in digestion, metabolism, and permeability of the intestinal mucosa to metabolically active molecules, and has emerged as a key factor in health and disease [36]. The role of the microbiome in CP/CPPS, a complex multifactorial disease, is becoming a focus of attention in clinical studies. Based on next-generation sequencing technologies, several studies have revealed potential associations between CP/CPPS and the composition of the microbiome in various types of samples, including feces, urine, semen, prostate massaged anterior urethral secretions, and expressed prostate secretions (EPS) [11, 37–39]. However, a few studies have explored the correlation between changes in gut microbiota and CP/CPPS, which is poorly understood. In addition, the use of gut microbiota for the diagnosis and treatment of lower urinary tract disorders is in the preliminary stages of research. In 2016, altered gut microbiota diversity in CP/CPPS patients was first reported in the U.S [11]. They demonstrated that the diversity of gut microbiota in CP/CPPS patients was significantly lower than that of controls, with significantly lower numbers of Prevotella than in controls, and that the level of isolation was sufficiently high to be used as a potential biomarker. Recently, a study [10] reported that compared with controls, Chinese CP/CPPS patients had a high representation of three bacteria and low levels of seven bacteria, presenting completely different dominant and subdominant intestinal microflora from those of US CP/CPPS patients, which predicts the geographic heterogeneity of CP/CPPS. However, there are still no studies that have established reliable diagnostic models for predicting CP/CPPS clinical outcomes based on specific gut microbiota species. The results of our study may provide some guidance for future related research.

Potential mechanisms underlying the link between gut microbiota and CP/CPPS are unknown. CP/CPPS patients often receive prolonged antibiotic therapy without evidence of culture or symptomatic response [40], which can alter the composition of the gut flora, and the disturbed gut microbiota often does not return to baseline levels after antibiotic discontinuation [41]. The common features of hyperalgesia and altered pain thresholds in patients with chronic pain also apply to patients with CP/CPPS [42], and changes in the gut microbiota may alter visceral nociception [43]. In addition, there is a link between the gut microbiota and the development of autoimmunity [44], and autoimmunity and dysfunctional immune responses are part of the pathophysiologic causes of CP/CPPS [45]. Communication between the gut and the central nervous system is bidirectional (gut-brain axis), in which the gut microbiota plays an important mediating role. The gut microbiota may affect the nervous system through various mechanisms [46], such as activation of the vagus nerve, modulation of the immune system, and production of neuroactive metabolites (e.g., short-chain fatty acids) [47]. Autonomic disorders are intricately linked to CP/CPPS. Dysregulation of the sympathetic and parasympathetic nervous systems can significantly impact organ function, serving as a catalyst for prostatitis. Concurrently, disturbances in sensory nerve activity contribute to the perpetuation of inflammation and pain. The phenomenon of central sensitization further exacerbates the condition by reducing pain thresholds and intensifying the perception of pelvic pain among individuals with CP/CPPS [48]. The association between the gut microbiota and CP/CPPS may indeed be influenced by psychological disorders. Individuals afflicted with CP/CPPS frequently grapple with psychological challenges, such as depression, stress, and catastrophizing [49]. The bidirectional dynamics of the gut–brain axis are crucial to understanding this relationship: stress has the capacity to modify the gut microbiome [50], and reciprocally, alterations in the gut microbiome can influence mood and behavior [51]. This intricate interplay underscores the complex connections between the gut microbiota and psychological factors in the context of CP/CPPS.

In this study, we failed to perform reverse MR analysis to assess bidirectional causation between gut microbiota and CP/CPPS, owing to the limited number of SNPs included in our analysis. Our study primarily focuses on elucidating the impact of gut microbiota on the risk of chronic prostatitis, aligning with the existing literature, notably the work of Liang et al., which underscores the role of Short-Chain Fatty Acid Propionate in Th17/Treg Cell Differentiation (52). However, conclusive evidence regarding whether chronic prostatitis influences gut microbiota requires further dedicated research. Future basic and clinical studies are imperative to unravel the complexities of how chronic prostatitis may influence gut microbiota, completing the bidirectional perspective.

This study still has some limitations. First, the results of the studies are all based on summary data from European populations, so there are some limitations to their generalizability. Second, due to the limitations of classifiers and sequencing depth, the gut microbiota can only be assessed for taxa above the genus level. Finally, we were unable to assess associations at the individual level due to the unavailability of genetic information from subjects. Future studies are needed in populations with multiple ancestry and large sample sizes.

## Conclusion

In summary, our analysis investigated the causal connection between 211 gut microbiota and CP. Notably, we identified 11 gut microbiotas as risk or protective factors for CP. Following FDR correction, Methanobacteria, Methanobacteriales, NB1n, Methanobacteriaceae, Odoribactergenus Odoribacter, and Sutterellagenus Sutterella still displayed significant causal association with CP. This study provides novel insights into the causal relationship between CP and gut microbial taxa. Our study had clinical significance as it opens up the new avenues and innovative strategies for the treatment of CP. However, further definitive experimental research is needed to elucidate the specific protective and detrimental mechanisms of these genera against CP.

### Supplementary Information

Below is the link to the electronic supplementary material.

Supplementary file1 (DOCX 358 KB)

Supplementary file2 (XLSX 249 KB)

## Data Availability

The data presented in this study have been deposited in publicly available datasets. These data can be accessed at the MiBioGen repository (https://mibiogen.gcc.rug.nl/) and the FinnGen repository (https://r9.finngen.fi/).
