# Supplementary material for: Causal effect of gut microbiota on the risk of prostatitis: a two-sample Mendelian randomization study
Source: Int Urol Nephrol. 2024 Apr 4;56(9):2839–50. doi: 10.1007/s11255-024-04020-w (PMC11322328; doi:10.1007/s11255-024-04020-w)
Supplement: Supplementary file 1 — Supplementary file1 (DOCX 358 KB) [file 11255_2024_4020_MOESM1_ESM.docx]

**Supplementary Figure S1.** Forest plots of the causal effects of gut microbiota on the risk of CP. (A) class Methanobacteria; (B) order Actinomycetales; (C) order Gastranaerophilales; (D) order Methanobacteriales; (E) order NB1n; (F) family Actinomycetaceae; (G) family Methanobacteriaceae; (H) genus Enterorhabdusgenus Enterorhabdus; (I) genus Erysipelatoclostridiumgenus Erysipelatoclostridium; (J) genus Odoribactergenus Odoribacter; (K) genus Sutterellagenus Sutterella.


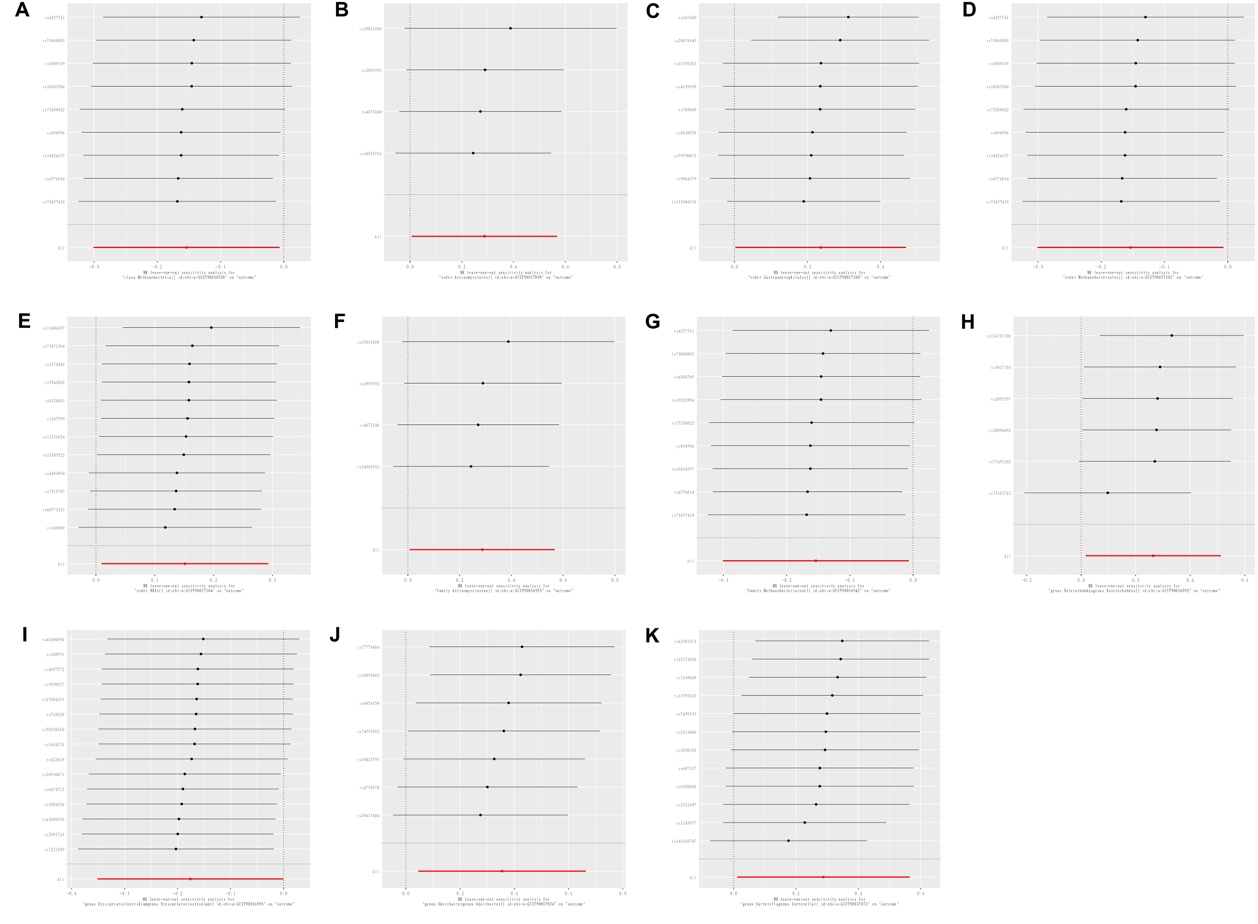


**Supplementary Figure S2.** Leave-one-out sensitivity analyses of the causal effects of gut microbiota on the risk of CP. (A) class Methanobacteria; (B) order Actinomycetales; (C) order Gastranaerophilales; (D) order Methanobacteriales; (E) order NB1n; (F) family Actinomycetaceae; (G) family Methanobacteriaceae; (H) genus Enterorhabdusgenus Enterorhabdus; (I) genus Erysipelatoclostridiumgenus Erysipelatoclostridium; (J) genus Odoribactergenus Odoribacter; (K) genus Sutterellagenus Sutterella.

**
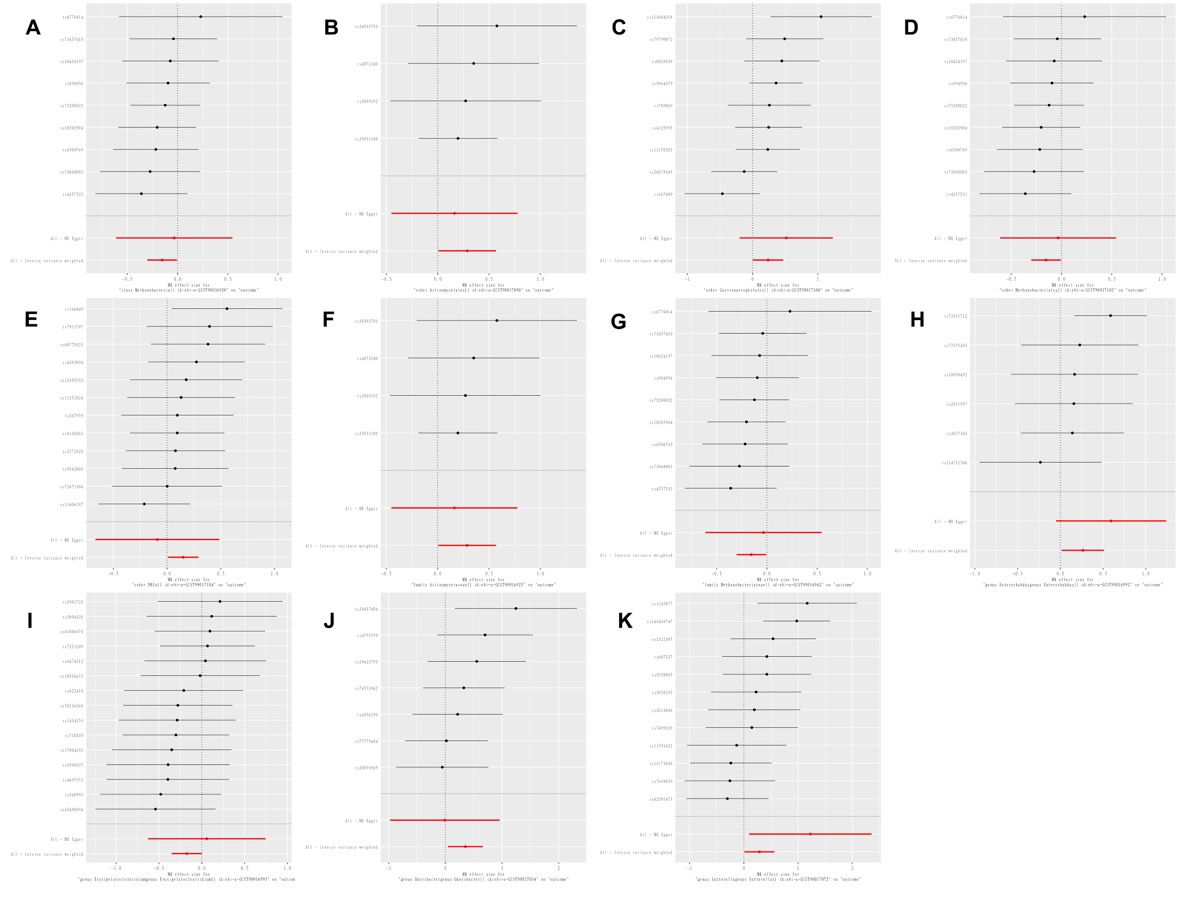
**
